# Analysis of Secondary Adhesion Wear Mechanism on Hard Machining of Titanium Aerospace Alloy

**DOI:** 10.3390/ma12122015

**Published:** 2019-06-23

**Authors:** Moises Batista Ponce, Juan Manuel Vazquez-Martinez, Joao Paulo Davim, Jorge Salguero Gomez

**Affiliations:** 1 Mechanical & Industrial Design Deptartment, University of Cadiz, Avda. de la Universidad de Cádiz 10, E11519 Puerto Real-Cádiz, Spain; juanmanuel.vazquez@uca.es; 2 Department of Mechanical Engineering, University of Aveiro, Campus Santiago, 3810-193 Aveiro, Portugal; pdavim@ua.pt

**Keywords:** titanium alloys, machining, turning, machinability, tool wear

## Abstract

Titanium alloys are widely used in important manufacturing sectors such as the aerospace industry, internal components of motor or biomechanical components, for the development of functional prostheses. The relationship between mechanical properties and weight and its excellent biocompatibility have positioned this material among the most demanded for specific applications. However, it is necessary to consider the low machinability as a disadvantage in the titanium alloys features. This fact is especially due to the low thermal conductivity, producing significant increases in the temperature of the contact area during the machining process. In this aspect, one of the main objectives of strategic industries is focused on the improvement of the efficiency and the increase of the service life of the elements involved in the machining of this alloy. With the aim to understand the most relevant effects in the machinability of the Ti6Al4V alloy, an analysis is required of different variables of the machining process like tool wear evolution, based on secondary adhesion mechanisms, and the relation between surface roughness of the work-pieces with the cutting parameters. In this research work, a study on the machinability of Ti6Al4V titanium alloy has been performed. For that purpose, in a horizontal turning process, the influence of cutting tool wear effects has been evaluated on the surface finish of the machined element. As a result, parametric behavior models for average roughness (Ra) have been determined as a function of the machining parameters used.

## 1. Introduction

Traditionally, the machinability concept involves the ability of materials for the development of removal processes to obtain a manufactured part [1], or the ability to be machined by using machine tools [2,3]. However, the initial concept is incomplete mainly due to the lack of definition of the variables of influence in the machinability and the experimental tests for a correct evaluation of the machinability properties. This problem was already described by Trent [4] studying the difficulty in the understanding of materials behavior during machining and the most relevant variables in the cutting processes. In order to obtain a more uniform machinability concept, Trent described five different criteria, the tool life, the material remove rate, the cutting forces, the surface finish and the chip shape. However, a general rate is not established for each specific criterion, therefore, it is not possible to make comparisons between different conditions of machinability, on the other hand, this consideration is specially based on economic-energy considerations, and currently the industry requires new criteria to evaluate the processes. This fact favors that the initially proposed considerations can be updated and extended to environmental and functional aspects [5]. In this sense, the study of the environmental impact of the process and the machined work piece features evaluated through the evaluation of the surface integrity is shown as an important research line [6]. Therefore, a relevant growth in the interest for dry machining was found by several authors in the last years [7,8].

Currently, using different methods of monitoring the machining process, the influence of different factors in this process can be accurately known. These elements can be considered as specific machinability criteria for each material and process. Special consideration is required in the case of materials with low machinability capacity such as titanium alloys, where the selection of the control parameters should allow knowing the range of parameters where the material can be machined under proper process conditions.

Another criterion to consider in the study of tool wear is the functional performance, related to the work behavior of the involved elements. Titanium alloys are widely used in the development of structural and strategic components of the aerospace industry [9]. The titanium alloys, and especially Ti6Al4V is considered a strategic material for the global market of the aerospace industry. In recent years, an important industrial interest in the use of this type of alloy has favored a significant increase in global consumption [9,10]. Due to the tolerance requirements of the aerospace industry, and the large volume of developed components with this type of materials, machining processes require accurate assembly conditions favoring the achievement of optimal dimensional and geometrical characteristics and excellent biocompatibility [11] for their use in work conditions. As a result, the machinability properties may be considered of strategic interest for the development of the manufacturing processes in the aerospace industry, especially due to the high influence of the process conditions on the surface integrity of the machined work-pieces [12,13].

Additionally, in order to reduce finishing procedures, maintaining micro-geometrical requirements of the surface, the study of the range of the parameters where the behavior of the tool reduces the development of a specific roughness is shown as a relevant aspect in the improvement of the manufacturing process performance [5]. 

However, when a titanium alloy is machined, the tool wear may imply an important problem and different ways are proposed to solve it. Lack of thermal conductivity of the substrate has been observed for titanium alloys, in many cases interfering with the machining process development [14,15,16,17] and being replaced in some cases by coatings such as PCD to improve the tool life [14]. With the aim to improve the economic performance and the lack of uniformity of a large volume of coated tools types, a generic uncoated tool was used to carry out the machining tests. In this way, the identification and characterization of the wear mechanisms that appears during the machining process is described [18,19,20,21,22,23,24].

The tool wear is related to the cutting speed. For titanium alloy, a cutting speed lower than 60 m/min is commonly used. Above of this cutting speed, in titanium, may be considered as high speed machining. In addition, cutting speeds higher than 150 m/min involves a quick high wear of the tool. This condition is considered ultra-high speed machining for titanium alloy, studied by Rashid et al. [18] and corroborate for Liang et al. [19] and Balaji et al. [25] for orthogonal cutting process. In an industrial process this consideration may be critical to improve the performance.

In this research, a study of the machinability of titanium alloy Ti6Al4V near the industrial environment is proposed based on the relationship between tool wear effects and micro-geometrical characteristics of machined parts in low, high and ultrahigh speed machining. With the aim of simplifying the study by reducing the control variables involved in the process and reduce the environmental impact, machining tests have been carried out by horizontal turning process in dry conditions.

## 2. Materials and Methods 

**Figure 1:**
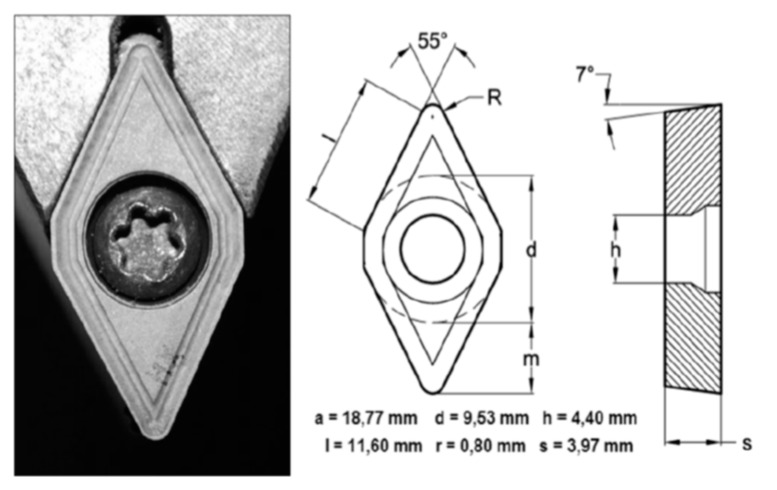
Cutting tool features.

Cylindrical bars of 150 mm length of UNS R56400 titanium alloy (Ti6Al4V) with an initial composition (wt%) shown in Table 1, were used as work-pieces to perform horizontal turning tests by means of a Kingsbury MHP 50 CNC Lathe Center (Kingsbury, Hampshire, UK). Cutting tools used were WC-Co uncoated inserts described by ISO ref DCMT 11T308, Figure 1. 

Three different types of dry turning tests have been carried out by varying machining conditions. A first set of the test has been performed to evaluate the wear effects on the tool surface and to analyze the appearance and evolution of adhesion wear mechanism, taking into account the analysis of the oxidation phenomena that takes place near the contact area, Table 2.

Additional tests were performed taking as reference the results of the main influence parameters with the aim to obtain a deeper understanding of the effects of the feed rate increase on the maximum speed studied (100 m/min). For this study, a cutting depth of 1 mm was kept and two higher different values of feed rate were selected (0.2 and 0.4 mm/rev).

Finally, taking the starting point the evaluations of the main influence parameters of the adhesive wear, the study of maximum tool life time for different machining process, including hard conditions were performed, from commonly used cutting speed ranges to ultrahigh speed. For this analysis, the machining conditions of Table 3 were used.

For the characterization of wear effects on the cutting tool an analysis of the main wear mechanisms that takes place in the process was carried out. Based on this consideration, the formation of adhered layers on the tool surface have been studied by means of scanning electron microscopy (SEM) (FEI Quanta 200, ThermoFisher Scientific, Hillsboro, OR, USA) and stereoscopic optical microscopy (SOM) techniques (SMZ-800, Nikon, Tokyo, Japan), taking in to account the oxidation processes on these layers. Oxidation phenomena mainly caused by high temperature of the contact area during machining stage have been identified using energy density spectroscopy (EDS) (Phoenix, EDAX, Mahwah, NJ, USA).

The use of a wide range of the machining parameters results in some different performances of the cutting effects. In this context, chip development can also be affected, giving rise to variations in the shape, length and thickness values.

Finally, as a consequence of geometrical variations of the cutting tool, mainly due to wear effects, and a wide range of chip growth behavior, the surface finish over machined work-pieces may be affected. Due to this fact, the evaluation of roughness of the parts surface was evaluated by a Hommel-Etamic T1000 Perthometer roughness measurement system (Jenoptik Group, Thuringia, Germany). It was analyzed in three different machining sections, initial, medium and final, and in each zone, a total of four roughness measurement was carried out in four different generatrices. 

In Table 4, a brief description of the experimental methodology is shown.

## 3. Results and Discussion

The analysis of wear effects on the cutting tools and machined parts caused by hard machining implies the study, identification and description of the most relevant mechanisms that takes place in the process. In this case, with adhesion being the main wear mechanism, the study of this mechanism needs a greater depth evaluation of the oxidation effects on the adhered layers. Additionally, the variation of the initial geometry and mechanical properties of the tool surface in the contact area during the machining stage may be the cause of the development of a wide variety of chip sections, with different features as length, width or thickness. Finally, the combination of tool modification by wear and the friction with different types of chip may affect the surface finish, in terms of roughness, of the machined parts, being relevant for specific applications.

### 3.1. Tool Wear Mechanism Characterization 

Wear by adhesive phenomena are shown as the main cause of modification in the initial geometry of the cutting area and shortening of the service life of the tools. This fact makes necessary a deeper analysis of the adhered layer development and their implications in the modification of the initial properties of the surface. Adhered material on the flank and rake face of the tool during the machining stage shows a direct influence of the aggressiveness of the cutting conditions, based on the feed rate, cutting speed and depth parameters [26].

**Figure 2:**
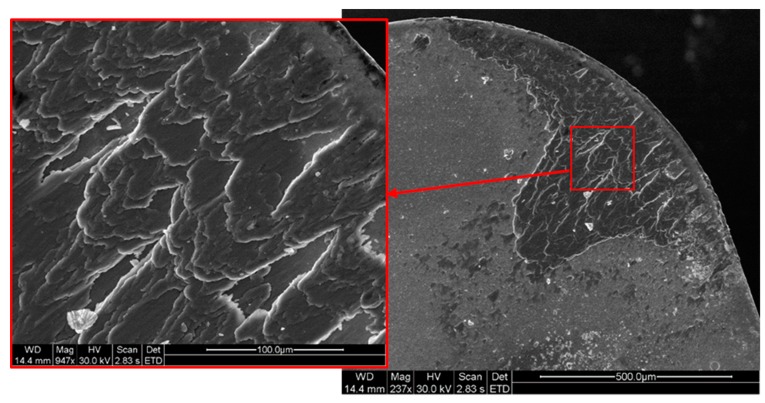
Built-up stratified layers on the rake face.

The evolution of the adhesion phenomenon starts with the growth of thin extruded layers of adhered material from the titanium alloy over the rake face-giving rise to primary and secondary built-up layers (BUL) and similar trends may be detected on the rake face, Figure 2. The thermo-mechanical effect is the main responsible of the adhesion phenomena on the tool surface, promoting the wear mechanism.

**Figure 3:**
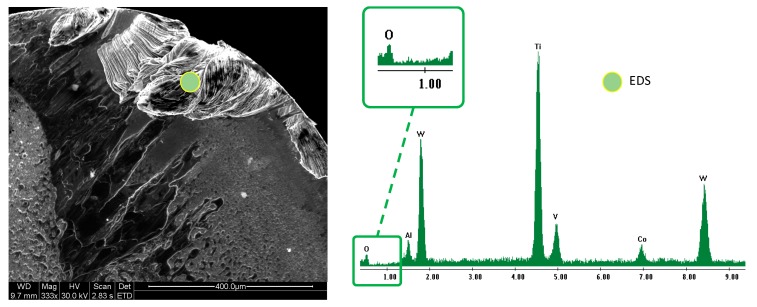
Oxidation phenomena on adhered material over rake face.

High temperature reached during the dry machining process near the contact area between the tool and work-piece, and the non-protective atmosphere where the test was performed, caused the oxidation of the extruded Ti6Al4V, Figure 3. 

**Figure 4:**
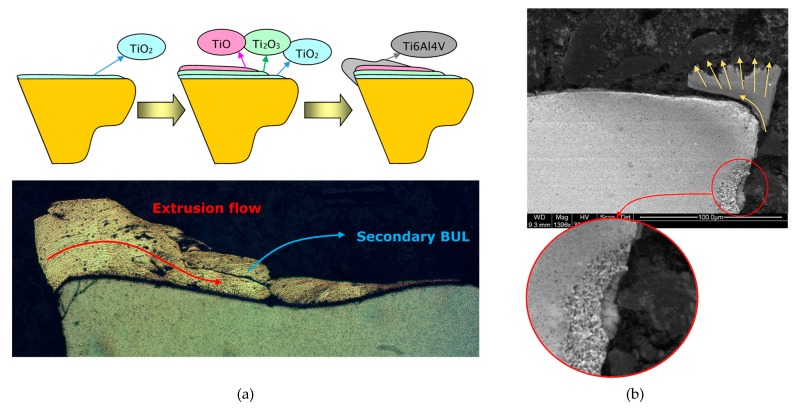
(**a**) Multilayer built-up layer (BUL) development process, (**b**) wear effects on clearance.

This fact results in significant changes on the contact behavior in the chip-tool interface, involving the development of a secondary BUL with a decrease of the oxidation effects from the tool surface to outer layers. Under this consideration, an influence may be confirmed of the titanium oxidized layers on the multilayer formation of the BUL and the built-up edge (BUE), Figure 4a. 

The development of BUE implies the increase of adhered multilayer secondary BUL to rake face being controlled by machining conditions. The increase of the cutting speed parameter results in the extrusion of the adhered material over farthest areas from the edge of the tool [27]. On the cross-section of the cutting tool two main phenomena can be detected. On the one hand, stratified layers of BUL extruded over the rake face allows to identify the flow lines of the material. On the other hand, the sliding process of the chip over the rake face implies friction phenomena and the corresponding temperature rise that promotes the adhesive wear mechanism. This fact is also associated with the development of wear effects on the clearance, as can be observed in Figure 4b, and according to [26].

Morphological changes mainly due to adhesion wear may cause a significant variation on the rake angle, giving rise to an increase of friction in the interface and conditioning the service life time of the tools. These geometrical variations also show critical implications on the process development, being relevant the damage consequences over the surface finish on the machined work-pieces and the shape and length of the chip.

### 3.2. Wear Evolution by Secondary Adhesion Mechanisms

**Figure 5:**
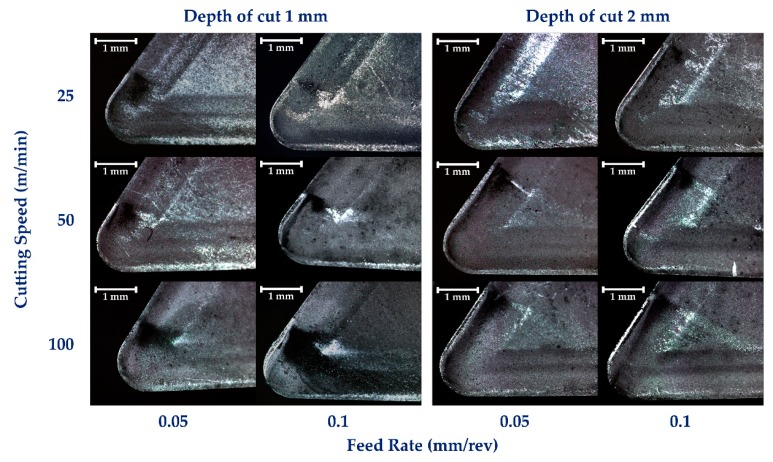
Wear evolution over the rake face.

The evaluation of wear effects on the uncoated carbide tools shows two different and representative regimes in the wear phenomena behavior based on the cutting speed parameter. For lower studied speeds (25–50 m/min) non-relevant changes can be detected for different values of the feed in mm/rev. Additionally, the increase on the cutting depth from 1 to 2 mm have not caused significant damages over the tool surface. However, the use of higher speed (100 m/min) involves variations in the wear behavior, giving rise to increase in the aggressiveness of the machining and highlighting the influence of feed in the cutting process, Figure 5. 

Under these considerations, abrasion wear marks are manifested by a dark area on the surface of the tool in Figure 5. This wear effect can be related to the friction between the chip and the rake face of the tool. Due to this abrasion mechanism, a drastic increase of the temperature in the surface is caused. As a consequence of high temperature and friction in the contact area, burn cases may occur giving rise to the darkening process of this area of the tool. Moreover, this increase in temperature can lead the diffusion of Ti in the tool matrix [24,28].

Due to the described abrasion mechanism, an important growth of the surface temperature occurs, being the cause in some cases of a burning process of the chip and darkening this area of the tool, according to [17]. This increase in temperature can lead Ti diffusion processes in the tool substrate [24,28]. As can be seen, this effect seems to increase as a function of the cutting speed and decrease as a function of feed parameter.

**Figure 6:**
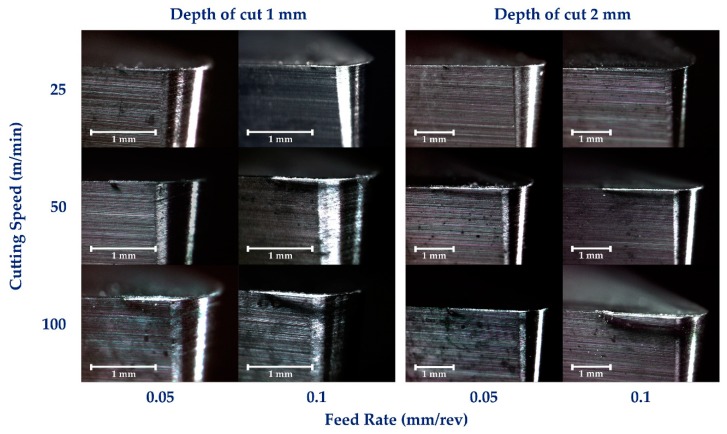
Wear evolution over the flank face.

This described behavior can also be observed over the flank face where the use of 100 m/min speed results in the development of color tone modification over the tool surface increases in the feed values, Figure 6. This fact confirms a more important influence of the feed rate for higher cutting speed according to [21,29]. In addition, color tone variation is related to the appearance of titanium oxide layers on the surface of the tool [30], induced by the high temperatures reached in the machining. Color tone variation is related to the secondary adhesion wear mechanism [14,21] and is manifested by the appearance of an oxide multi-layer on the tool that favors adhesion of the alloy material on the rake face and tool edge, developing the BUL-BUE morphologies [14,21,26]. The development of oxidation layers favors the modification of the surface properties of the cutting tool in the contact area.

**Figure 7:**
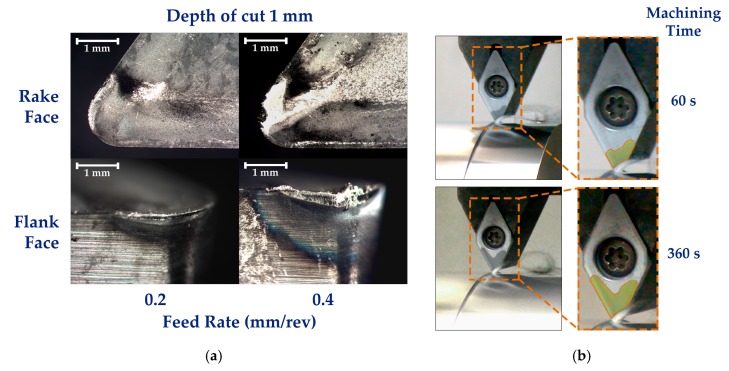
(**a**) Wear effects on flank and rake face of the turning tool for different feed rate using 100 m/min cutting speed; (**b**) oxidative layer growth over tool surface during machining process.

As described above, feed rate variations carried out on the higher studied cutting speed (100 m/min) show an important influence in the wear evolution of the uncoated tool. In this sense, with the aim to obtain a better understanding of this situation, a deeper study was performed on this behavior from the most aggressive machining conditions setting initially. This study has evaluated the wear effects in 1 mm depth of cutting and a range of feed rate values of 0.2 and 0.4 mm/rev by using a cutting speed of 100 m/min. Under these conditions, a severe growth of the wear effects have been detected, and adhesion mechanisms are shown as the most adverse phenomena against the tool life time, Figure 7a.

The oxidative phenomena that takes place under these machining conditions results in a quick darkening of surface color tone of the uncoated tool. The thermal oxidative layer growth along the tool surface as a function of the machining time, mainly due to high temperatures reached in air atmosphere (without protective environment), can be observed in Figure 7b.

### 3.3. Influence of Secondary Adhesion Wear on the Service Tool Life of the Cutting Tool

Adhesive wear on the cutting tool surfaces may be the main cause to reduce the life time under working conditions. The use of aggressive machining conditions as well as the lack of lubricating or cooling fluids accelerates the wear effects on the tool, and results in the most severe conditions that imply critical failures. With the aim to understand the influence of wear on the tool life time, turning tests were conducted using cutting speed as reference parameter, due to their influence in adhesive wear mechanisms. 

**Figure 8:**
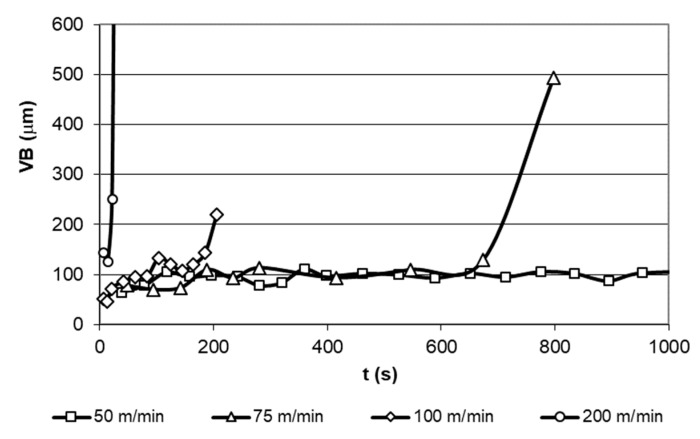
Life time of the tool as a function of the cutting speed.

Flank wear (VB) was determined on a range of cutting speed from 50 to 100 m/min reaching a maximum duration of the machining process previous to the failure of the tool. As can be seen in Figure 6, the test for 50 m/min cutting speed did not show significant wear damages, being interrupted after 30 min. Additionally, the ultra-high speed test (200 m/min) was carried out to evaluate the system behavior on maximum aggressive conditions and ensure a direct influence of the cutting speed in the life time of the cutting tool. As can be expected, the life time of the tool, based on the VB analysis, is strongly affected by cutting speed, reaching a minimum time of 29 s from the start of the machining process to the tool failure for a cutting speed of 200 m/min, Figure 8 according to [31].

The VB evaluation on different cutting speed have allowed to estimate the coefficients for the life time of the tool as a function of the speed through the Taylor tool life equation:

(1) $$T=C\cdot v^{k}$$

where *T* is the time life of the tool in minutes and *v* is the cutting speed (m/min).

By means of the Taylor equation on the experimental data, a new expression was obtained where the k coefficient allows corroborating the results with other authors’ researches [10,18,25,28] on carbide tools and according to the ISO 3685 standard.

(2) $$T=58.12\cdot 10^{6}\cdot v^{-3.58}$$

As described above, the tool life end in previous experimental tests results in a critical failure that could be the cause of significant damages on the different elements involved in the machining process. This critical failure implies a drastic variation of the cutting edge geometry, giving rise to the modification of the surface finish, in terms of roughness, of the work-piece. Additionally, this edge variation in combination with adhesive wear effects, causes relevant changes in the rake angle, changing the initial thickness, shape and length of the chip. 

**Figure 9:**
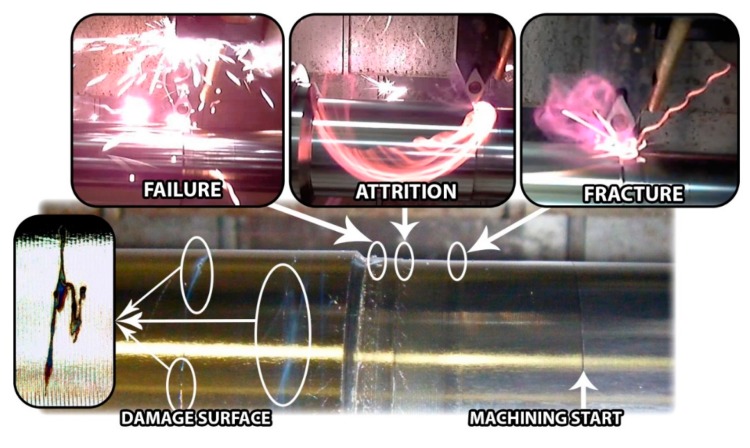
Tool wear evolution in the last stage of the life time.

On the final stage of the machining, near critical failure, some specific phenomena were detected on the development of the tests, corresponding to visual and profilometry analysis of the machined surface, Figure 9. 

After the stationary stage of the machining process, the critical failure phenomenon starts with the increase of the wear damage over the clearance, promoting a growth in the contact interface between the cutting tool and work-piece. This effect is generally associated with the fracture/attrition of small fragments of the tool and an important rise of the friction between contact elements. In most cases, an increase of friction implies an increase of the temperature, reaching burning situations of the chip. These aggressive conditions involve severe adhesion effects and the fracture of the tool through the detachment of the adhered material and removing particles from the carbide surface.

### 3.4. Tool Wear Effects on the Surface Finish of Machined Parts

**Figure 10:**
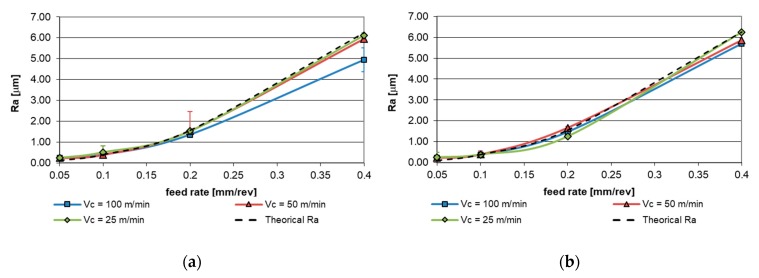
Evolution of the roughness average (Ra): (**a**) Cutting depth 1 mm; (**b**) cutting depth 2 mm.

In order to evaluate the impact of the tool wear effects on the surface finish of the machined work-pieces, roughness measurements have been performed over the turned sections. Figure 10 shows the evolution of the roughness as a function of the feed for the three cutting speed studied compared with the theoretical value obtained by the classical expression exposed in Equation (3). For the measurement of surface finish, roughness average (Ra) has been selected as a control parameter to quantify the variations caused by the wear effects.

(3) $$Ra_{t}=\frac{a^{2}}{32\cdot R}\cdot 1000$$

An important dependence was detected on the surface finish, in terms of Ra, with respect to the feed. This fact is mainly due to the formation of specific microgeometry relative to the displacement of the tool on the machined surface. Furthermore, lower dependence of Ra on the cutting speed is observed, which increases for higher values of feed. This dependence decreases for 2 mm depth of the cut.

In order to clarify this effect, parametric models have been established for each value of the cutting depth used. Equation (4) corresponds to the depth of the cut of 1 mm and Equation (5) is related to 2 mm of depth.

(4) $$Ra_{p1}=30.649\cdot f^{1.554}\cdot \;v^{-0.109}$$

(5) $$Ra_{p2}=19.668\cdot f^{1.566}\cdot \;v^{0.011}$$

As can be observed in previous equations, the exponent of the feed (*f*) is close to a constant value, ensuring a similar behavior for 1 mm and 2 mm depth, however, in the case of the cutting speed (*v*) relevant variations in the exponent have been detected between values of depth. 

When the depth of the cut is included in the surface finish behavior expression, the feed exponent maintaining similar values as previous equations show the consistency of the proposed model.

(6) $$Ra_{p}=23.662\cdot \;f^{1.561}\cdot \;v^{-0.041}\cdot \;p^{0.012}$$

However, the tool wear may affect the surface quality of the machined parts. In this way, it is known that the wear behavior is not linear, showing a steadily increase until reaching a rapid growth point. For this reason, the proposed models described in Equations (4) to (6) have been determined for an intermediate machined section where the wear behavior could be considered in a stationary stage. Furthermore, previous studies have determined an effect that reduces the surface quality of the machined parts in a stable wear section [32,33], as in the aluminum case [34,35,36,37]. This effect is related to the accumulation of adhered material into the tool, which causes a reduction in the values of the roughness, according to [36,37].

## 4. Conclusions

A direct relationship has been detected, in constant machining length conditions, between the tool wear and cutting parameters. This behavior is mainly related to thermo-mechanical effects during machining process.

The feed is shown as a control parameter for the adhesion mechanisms and cutting speed for the abrasion wear phenomena. By increasing these parameters, the wear mechanism effects also increase, maintaining this trend for all the studied depth of the cut.

Regarding the surface finish of the machined parts, the roughness, in terms of average roughness parameter, of the work-pieces show a direct dependence with the feed parameter. This fact is mainly due to the micro-geometrical effects of the cutting tool displacement, therefore, the experimental Ra obtained is relatively closed to the theoretical Ra based on the geometric model.

An increase in the cutting speed results in variations between theoretical and experimental values of the roughness of the machined parts. The lack of concordance between theoretical and experimental models can be associated with the appearance of the tool wear in the flank face for high values of cutting speed.

Through increases in 50 m/min of cutting speed, a significant reduction in the life time of the tool have been detected. This effect may be produced especially by a rapid growth of the flank wear of the tool. This behavior has as a result that, in the case of higher cutting speeds, the tool life is greatly reduced and critical failures can be caused.

A near exponential behavior has been detected between the increase of the cutting speed and the toll life, showing an approximately value of 75%–90% reduction for the tool life under high speed conditions (>50 m/min).

## Figures and Tables

**Table 1:** Composition (wt%) of Ti6Al4V titanium alloy.

Al	V	Fe	C	O	N	H	Ti
6.29	4.04	0.16	0.008	>0.05	>0.05	>0.05	Rest.

**Table 2:** Machining conditions for the evaluation of adhesion wear evolution.

Cutting Speed (m/min)			Feed Rate (mm/rev)		Cutting Depth (mm)	
25	50	100	0.05	0.1	1	2

**Table 3:** Machining parameters for the evaluation of tool life time under standard and hard conditions.

Cutting Speed (m/min)				Feed Rate (mm/rev)	Cutting Depth (mm)
50	75	100	200	0.2	1

**Table 4:** Experimental methodology followed in this research.

Study	Method	Objective
Tool wear mechanism characterization	Single test SEM/EDS analysis Cross-section of the tool evaluation	Characterization of tool wear mechanism for Ti6Al4V
Wear evolution by secondary adhesion mechanisms	Parametric test: f(S,f,d) SOM analysis	Evolution of the tool wear as a function of cutting parameters
Influence of secondary adhesion wear on the service tool life of the cutting tool	Tool life test: f(S) SOM analysis Tool wear measurement	Evolution of the tool wear regarding cutting time and establishment the tool life.
Tool wear effects on the surface finish of machined parts	Parametric test: f(S,f,d) Surface roughness analysis	Relation between cutting parameters and roughness as a function of tool wear.
